# Diagnostic value of diffusion-weighted MR imaging in thyroid disease: application in differentiating benign from malignant disease

**DOI:** 10.1186/1471-2342-13-23

**Published:** 2013-07-30

**Authors:** Yingwei Wu, Xiuhui Yue, Weiwen Shen, Yushan Du, Ying Yuan, Xiaofeng Tao, Cheuk Ying Tang

**Affiliations:** 1 Radiology Department, East Hospital, Tongji University, school of Medicine, Shanghai 200120, China; 2 Radiology Department, Shanghai People’s Ninth Hospital, Shanghai Jiaotong University, School of Medicine, Shanghai 200011, China; 3 Radiology Department, Shanghai Changzheng Hospital, Shanghai, China; 4 Radiology Department, Mount Sinai School of Medicine, New York 10029, USA

**Keywords:** Thyroid lesions, Diffusion weighted imaging, ADC mapping

## Abstract

**Background:**

Fine needle aspiration biopsy is usually performed to evaluate thyroid lesions. The purpose of this study was to evaluate the usefulness of diffusion weighted imaging to differentiate malignancy of thyroid lesions.

**Methods:**

The study was approved by ethics committee of Shanghai Changzheng Hospital.Forty-two patients, 10 men and 32 women (range: 20–72 years, mean age 42.4 years) with thyroid lesions were included in the study. Routine neck MR and diffusion-weighted MR imaging was performed using multiple b-values. ADC values were computed for the different b-values. Histological results of the thyroidectomy samples were obtained for all the patients. ADC values of benign and malignant thyroid lesions were compared with the pathology results. Logistic regression analysis was used to detect independent parameters for differentiating benign and malignancy of lesions.

**Result:**

Based on the histology results there were 28 benign and 14 malignant cases. The difference of ADC value between benign and malignant thyroid lesions was significant for ADC values obtained using b-values of 0 and 300 s/mm^2^ (p < 0.001). The ADC values were significantly higher in benign lesions (benign ADC: 2.37 ± 0.47 × 10-3 mm^2^/s vs. malignant: 1.49 ± 0.60 × 10-3 mm^2^/s). ADC values obtained with b-values of 0 and 300 mm^2^/s and max nodular diameter was regarded as the two most discriminative parameters for differentiating malignancy. Using the pathology results as a standard reference, area under ROC curve was found to be 0.876 for an ADC cutoff value of 2.17 × 10-3 mm^2^/s that corresponded to an acquisition with b-values of 0 and 300 mm^2^/s.

**Conclusion:**

Diffusion-weighted MR imaging is a promising non-invasive method to differentiate malignancy in thyroid lesions.

## Background

Incidence and prevalence of thyroid disease has seen a great increase in recent years. An increasing incidence of thyroid cancer has been reported worldwide [1-3]. The reported incidence of thyroid cancer is up to 0.07% over the past 30 years [4]. 44,670 new cases and 1,690 deaths were attributed to thyroid cancer in 2010 [5]. Fine needle aspiration biopsy (FNAB) is regarded as the standard reference for diagnosis, but it has been reported before that FNAB results may mimic some other diseases [6-8]. Ultrasound is the most common non-invasive and sensitive diagnostic imaging modality for diagnosing thyroid lesions, but there are still no reliable criteria for distinguishing benign from malignant lesions. In addition, it is difficult to access the malignancy of the nodule when it is large or multinodular [9]. Despite tremendous improvement in diagnostic techniques such as ultrasound, radionuclide imaging and CT, there is still a big challenge to have a non-invasive and reliable technique to differentiate benign from malignant lesions. Recent developments in MRI may show that some MR protocols are of diagnostic value for these types of lesions [10]. Routine T1- and T2-weighted MR imaging can provide information on the location and size of thyroid lesions. But these protocols still don’t have the specificity for distinguishing benign from malignant nodules or assessing the functional status of these thyroid nodules.

Diffusion-weighted MR imaging (DWI) is an emerging technique for central nervous system (CNS) diseases. DWI is sensitive to changes in the microstructural organization of tissue that may affect water diffusion. It has been used in various forms to evaluate head and neck tumors [11-13]. The Apparent Diffusion Coefficient (ADC) value, a metric obtained from DWI scans, could be a quantitative parameter for distinguishing malignant tumors from benign lesions.

In our current study, we compared ADC values of thyroid lesions with their pathology reports in order to evaluate its usefulness for distinguishing malignancy.

## Methods

### Patient selection

Between July 2007 and Jan 2011, 42 patients (10 male; 32 female) with palpable or ultrasonography determined thyroid nodules were prospectively enrolled and patient consent was obtained. The study was approved by ethics committee of Shanghai Changzheng Hospital. Mean age of those patients was 42.9 yrs (range 20-72 yrs). Routine neck MR imaging and diffusion-weighted MR imaging was performed for each patient. All patients enrolled for this study have been scheduled to have thyroidectomy within 2 weeks. Thyroidectomy was performed afterwards and pathology results were obtained for each patient. Conventional histology revealed that 28 patients had benign lesions, including thyroid adenoma (N = 20), nodular goiter (N = 5), and Hashimoto's thyroiditis (N = 3) while 14 patients were found to have malignant lesions, including thyroid papillary carcinoma (N = 10), follicular thyroid cancer (N = 3), atypical hyperplasia (N = 1). Thyroid lesions less than 1 cm were excluded because of diagnostic difficulties in evaluation.

### MRI protocol

MR scanning was performed on GE Signa HD 1.5 T MR scanner (GE healthcare, USA). Routine MRI examinations were done with the following parameters: fast spin echo FSE scanning for neck, axial T1WI (TR = 520 ms, TE = 14 ms), T2WI (TR = 3500 ms, TE = 95 ms) and coronal T2WI (TR = 3000 ms, TE = 85 ms). Thickness = 4 mm, Spacing = 1 mm. FOV = 14 cm × 14 cm, Matrix = 320 × 256, NEX = 4.

Diffusion Weighted MR Imaging (DWI) was acquired using four different b factors (0, 300, 500, and 800 s/mm^2^) in a STIR fat suppressed SE-EPI sequence. All DWI scans were acquired with the same parameters:TR = 3000 ms, TE = 60 ms, Matrix = 96 × 128, thickness - 4 mm and 6 averages were obtained. Total scanning time of about 10 minutes for the DWI scan.

### Image post processing

Using the post processing software Functool from GE, ADC maps were automatically generated for each of the b factors. ADC values were extracted from ADC maps. Circular ROIs (regions of interests)with an area of 1 cm^2^ were carefully placed on the lesions (areas of necrosis, hemorrhage, calcium and cyst formation were excluded). ROIs were placed on one ADC map and then propagated to the other 2 by two experienced radiologists blinded to each other’s delineations. To minimize noise, each radiologist made three measurements for each lesion and the mean ADC value was recorded. Interrater reliability of the ROIs ADC values had ICC > 0.87. Final ADC value was determined by the mean values from two raters.

### Analysis

ADC maps were computed for each of the b-values used in the DWI protocol. This was done automatically for the b-factors of 300 s/mm^2^, 500 s/mm^2^ and 800 s/mm^2^. ADC values were extracted from the ADC maps. Statistical analysis was performed using the Statistical Package for the Social Sciences for Windows (SPSS, Chicago, Ill). All ADC data were recorded in Mean ± SD (×10^-3^ mm^2^/s) form. Mann–Whitney U-test was performed to compare the quantitative ADC value of benign and malignant thyroid nodules. A value of p < .05 was considered significant. In addition, receiver operating characteristic (ROC) curve was constructed to determine a cutoff value for differentiating benign and malignant thyroid lesions. Receiver operating characteristic (ROC) curves were constructed and areas under the ROC curve (Az) were computed using MedCalc version 10.2.0.0 (http://www.medcalc.com/).

## Results

MRI results revealed that among those 42 cases of lesions in the thyroid, 10 were located in the left lobe of thyroid gland, 16 of were in the right lobes, 14 had lesions on both lobes and 2 lesions were in thyroid isthmus. For those with lesions on both lobes, one was randomly chosen (matched with pathology) for ADC measurements. Mean major diameter of the benign and malignant lesions were 3.63 cm and 2.55 cm respectively. Historical findings confirmed there were 28 benign cases and 14 malignant lesions. Independent of malignancy, thyroid lesions occurred more frequently in female than in male (Table 1).

**Table 1:** Historical results and patient characteristics

***Histological categories***	***Number *(%)**	***Mean age *(*yrs*)**	***Female *(%)**	***Mean maximum diameter *(*cm*)**
**Benign**	28(66.7)	41.7	21(75)	3.63 ± 0.37
Thyroid adenoma	20(47.6)	42.6	15(53.6)	3.53 ± 0.25
Nodular goiter	6(14.3)	39.3	5(17.9)	3.95 ± 0.65
Hashimoto's thyroiditis	2(4.8)	35.6	1(3.6)	3.71 ± 0.11
**Malignant**	14(33.3)	45.3	11(78.6)	2.55 ± 0.41
Thyroid papillary carcinoma	11(26.2)	41.9	9(64.4)	2.73 ± 0.54
Follicular thyroid cancer	2(4.8)	49.1	1(7.1)	1.7 ± 0.13
Atypical hyperplasia	1(2.4)	45	1(7.1)	2.3

### Parameters for differentiating lesion malignancy

**Figure 1:**
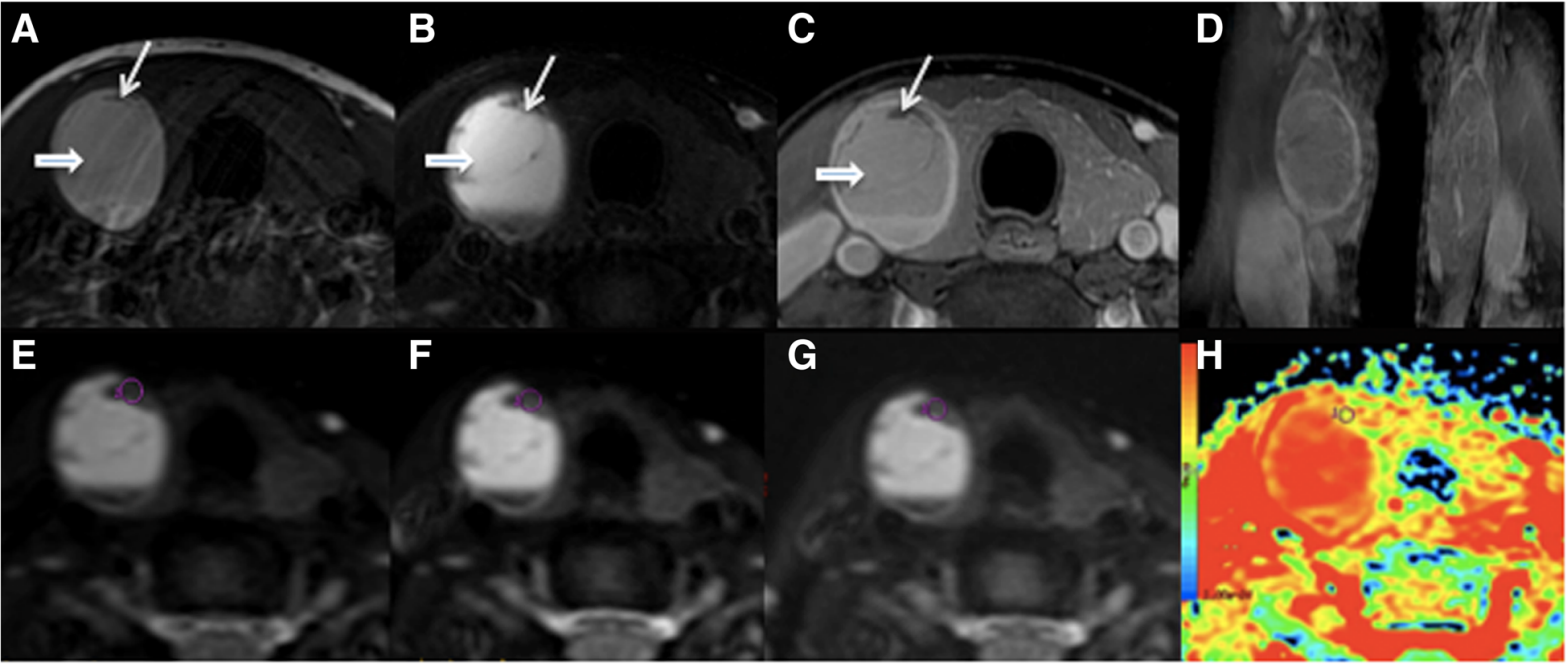
**A 44-year-female patient with right lobe thyroid adenoma; (A-C) Non-contrast and contrast transversal images showed a hemorrhage in the right lobe (short thick arrow).** Long thin arrow shows the lesion. **(D)** Coronal images showed the well-circumscribed lesion with homogenous enhancement. **(E**-**G)** showed ADC value obtained from ADC map with b factors of 0, 300, 500 and 800 s/mm^2^, respectively. ROIs were placed in the lesion at right upper area to avoid the hemorrhage area. **(H)** ADC map generated at b-factor of 300 s/mm^2^.

**Figure 2:**
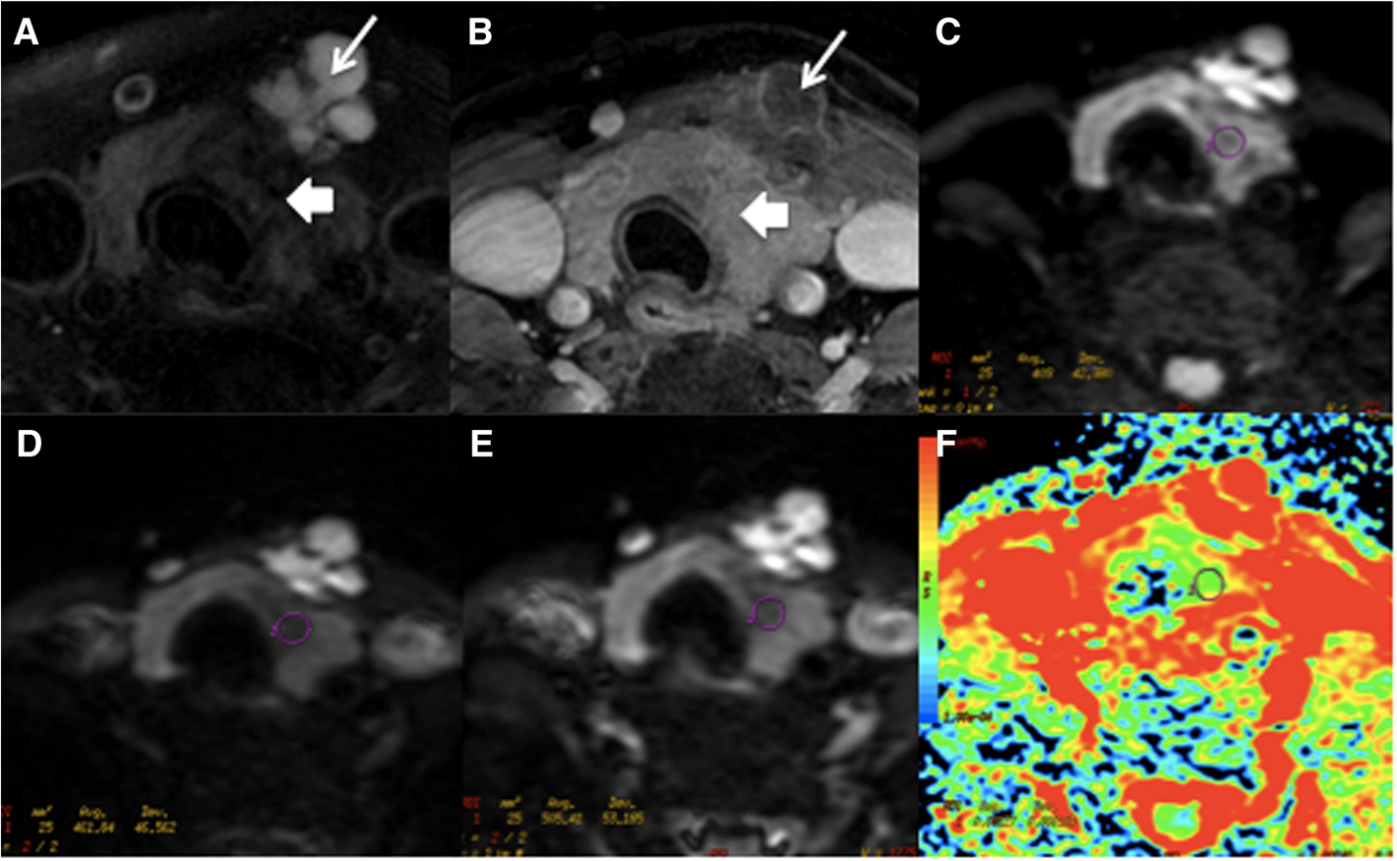
**A-36-year-female patient with thyroid papillary carcinoma at left lobe and isthmuses is shown. (A**-**B)** Non-contrast and contrast transversal images showed abnormal signal at left lobe and isthmus with multiple cysts (long arrows). **(C**-**E)** showed ADC value measured from ADC map with b factors of 300, 500 and 800 s/mm^2^, respectively. **(F)** ADC map generated at b-factor of 300 s/mm^2^.

Using histology results to group the ADC values we found that the ADC values were significantly different (p < 0.001) between benign and malignant lesions for the ADC values computed for b = 300 s/mm^2^. Mean ADC value for benign group (Figure 1) is much higher than that for malignant group (Figure 2), with the values of 2.37 ± 0.47 × 10^-3^ mm^2^/s and 1.49 ± 0.60 × 10-3 mm^2^/s respectively. However, no significant difference was observed between the two groups for the ADC values obtained using b = 500 s/mm^2^ or 800 s/mm^2^ (Table 2). Sensitivity, specificity and AUC were also compared among three different b-factors. Highest sensitivity and AUC was observed at b-factor of 300 s/mm^2^ (Table 3).We also selected four variables (age, sex, ADC value, max nodular diameter) as independent variables to perform logistic regression analysis. Odds ratios (and 95% CIs) based on the logistic regression model were also calculated. The results obtained from the logistic regression model showed that ADC values obtained with a b factor of 300 s/mm^2^ (OR = 4.76, p <0.001) and max nodular diameter (OR = 3.22, p = 0.034) were the two most discriminative independent variables for differentiating between benign and malignant lesions (Table 2).

**Table 2:** Association between parameters and disease property

	**Benign group (n = 28)**	**Malignant group (n = 14)**	P value*	**OR**	**95% CI**
**Age (yrs)**	41.7	45.3	0.79	0.62	0.26, 2.59
**Sex (Female %)**	21(75)	11(78.6)	0.61	0.50	0.16, 1.54
**Max nodular diameter (cm)**	3.63 ± 0.37	2.55 ± 0.41	0.034	3.22	1.27, 6.76
**ADC values [mm**^**2**^**/s]**					
**b-0,300**	2.37 ± 0.47	1.49 ± 0.60	<0.001	4.76	1.56, 9.89
**b-0,500**	1.87 ± 0.25	1.61 ± 0.45	0.138	1.01	0.3, 1.81
**b-0,800**	1.68 ± 0.25	1.41 ± 0.34	0.059	0.091	0.03, 0.73

*p value reflected from logistic analysis.

**Table 3:** Sensitivity, specificity and AUC of the use of mean ADC value as calculated based on 3 different b-values for differentiating benign from malignant thyroid lesions

	**b = 0,300**	**b = 0,500**	**b = 0,800**
AUC	0.88(0.77–0.97)	0.63(0.47–0.77)	0.63(0.46–0.77)
Criterion [10^-3^ mm^2^/s]	>2.17	>1.74	>1.65
Sensitivity %	76.5	67.9	53.6
Specificity %	100	64.3	71.4

### ROC analysis

**Figure 3:**
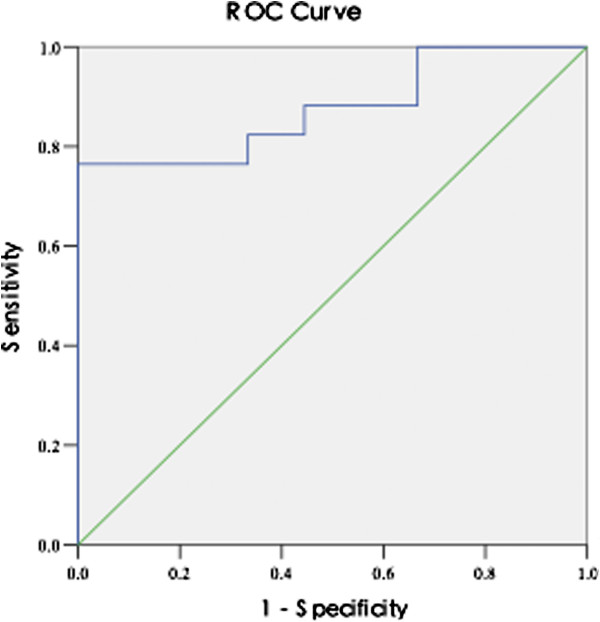
Receiver operating characteristic (ROC) curve with the ADC value (computed from DWI with b = 0 and 300 s/mm^2^) used for differentiating benign from malignant thyroid lesions.

Furthermore, a Receiver Operator Characteristic (ROC) curve was computed for the ADC values obtained from DWI scans using b = 300 s/mm^2^. We determined a cutoff point from the ROC curve that would differentiate benign from malignant lesions. The area under the ROC curve was 0.876. When we selected a cutoff ADC value of 2.17 × 10^-3^ mm^2^/s, sensitivity and specificity was found to be 76.5% and 100% respectively (Figure 3).

## Discussion

To date, only a few studies have focused on the use of DWI in evaluating thyroid disease. Tezuka [14] et al. used both routine T1WI, T2WI and DWI techniques to diagnose thyroid diseases (Graves’ disease, sub-acute thyroiditis and Hashimoto's disease). Although no obvious difference using T1WI and T2WI were detected on those thyroid diseases, ADC values of thyroid lesions of Graves’ disease was found much higher than that of sub-acute thyroiditis or Hashimoto's disease. An ADC value of 1.82 × 10^-3^ mm^2^/s was suggested as the cutoff point to diagnose Graves’ disease (Sensitivity 75% and Specificity 80%). DWI has emerged as a potential new noninvasive technique that can provide more quantitative information on thyroid lesions and help make clinical differential diagnosis.

### MR technique

Diffusion weighted MR imaging can be done with single shot or multi-shot. Wang and Adel Razek [11,15] applied single-shot multisection echo-planar technique, using b values of 0, 500 and 1000 s/mm^2^ respectively. This sequence is characterized by a train of gradient echoes with a short echo time image and good signal-to-noise ratio (SNR), but it was associated with more magnetic susceptibility artifacts. Tezuka [14] et al., used a fast spin echo sequence for diffusion-weighted imaging to avoid magnetic susceptibility artifacts, but not only was the imaging time increased but the SNR was also decreased.

In this study, we used a STIR (Short TI Inversion Recovery) fat suppressed SE EPI (Spin-Echo Echo Planar Imaging) sequence. STIR is a very robust fat suppression technique with minimal artifacts. It has low sensitivity to magnetic field inhomogeneities or susceptibility effects and thus improves image quality. It also produces a slight T1-weighted background suppression but does not affect the accuracy of the images. EPI is the fastest acquisition method and it only requires 30 ms ~ 100 ms to collect one image which helps in reducing most physiological movement artifacts [16]. Furthermore, short TE time of 60 ms was used to reduce T2* effect in our study. Thus, using this sequence we were able to obtain more accurate tissue ADC values.

The b-factor in the DWI was an important factor for image quality. We obtained diffusion-weighted MR images with different b factors simultaneously to avoid misregistration in computing the different ADC values. Higher b-values produce more diffusion weighting and therefore higher contrast between thyroid lesions and normal tissue. However, higher b value also leads to increased signal attenuation and usually required more averages to compensate for the SNR. Higher b-values also produce more susceptibility distortions and could increase the noise in the DWI images because the distortions are different depending on the gradient directions. In addition, different tumors within different organs or tissues may be more sensitive to different b-values. In this study, we applied an SE EPI diffusion imaging with b-factors of 300, 500 and 800 s/mm^2^. Our results showed that a b-factor of 300 s/mm^2^ was sufficient to obtain high quality ADC values and it was also the one with had a high sensitivity and accuracy for differentiating benign and malignancy. These results are consistent with Bozgeyik’s [17] results.

### Pathological mechanism of ADC values in thyroid lesions

In our study we were able to use the most reliable reference for malignancy which was based on the histopathology of a thyroidectomy. Our results showed a significant difference in the ADC value between benign and malignant thyroid lesions, with ADC values in benign lesions being higher than malignant lesions (2.37 ± 0.47 × 10^-3^ mm^2^/s vs. 1.49 ± 0.60 × 10^-3^ mm^2^/s) at the b factor of 300 s/mm^2^. These results are consistent with other similar studies [17-19]. Diffusion weighted imaging provides more information on the microstructure of tissues and their physiological processes. Changes in the distributions of intracellular organelles and macromolecules in the tissues affect the random motion of water protons. ADC values of tissues vary according to its cellularity and histopathology. Anderson [20]’s study showed that malignant lesions in thyroid glands was characterized by compact cellularity which increased the nucleocyte-cytoplasmic ratio (NCR). These microscopic pathological changes results in the reduction of extracellular space which limits diffusional motion of water protons. Reduced ADC values are presented as high signal on DWI sequences.

Abdel’s [21] study demonstrated that when selecting ADC value of 0.98 × 1.0^-3^ mm^2^/s as a cutoff point to differentiate benign and malignant lesions, the sensitivity and specificity was 97.5% and 91.7%, respectively. The accuracy was up to 98.9%. In this study a single pair of b-values (0, 1000 s/mm^2^) were studied. Although we also looked at the ADC values obtained using higher b-values (0,800 s/mm^2^), we did not detect any useful threshold at the higher b-values. Abdel’s study included a variety of cervical lymph nodes whereas our study focused on the thyroid. Lesion heterogeneity might have accounted for the differences in sensitivity. It was also suggested that the ADC values of benign thyroid nodule may vary according to the complex composition within the nodule (colloid, tiny necrosis and cystic change, hemorrhage, fibrosis and calcium). ADC values were highest in thyroid cysts since it contained colloid cyst made of serous or concentrated thyroglobulin. Conversely, increasing NCR and grit-like calcification mainly lead to a decrease of ADC values in papillary thyroid carcinoma.

Bozgeyik [17] also studied thyroid nodules with DWI at lower b-values. Bozgeyik [17] also found that a b-factor of 300 s/mm^2^ was the most useful for differentiating benign or malignant lesions. In Bozgeyik’s study 3 b-value pairs were used to computed the ADC (0, 100 s/mm^2^), (0, 200 s/mm^2^) and (0, 300 s/mm^2^). We expanded on these results and investigated ADC values obtained using larger b-values. Our sample includes about 33% malignancy rates whereas in Bozgevik’s study less than 5% of the nodules were malignant. In their study, malignancy was determined using FNAB whereas in our study we have used pathology results from thyroidectomy, a more accurate reference. Erdem [19] also suggested that tiny calcification was closely correlated with the reduction of ADC values in thyroid malignancy.

However, in Weidekamm’s [22,23] study, the opposite result was reported. ADC values were predominantly higher in malignant thyroid nodules than that in benign nodules with values equal or more than 2.25 × 10^-3^ mm^2^/s. The higher ADC values may be due to the over production of thyroprotein follicle in malignant thyroid nodules which do not restrict the diffusion of the water protons.

ADC values has been used as a quantitative parameter for differentiating benign and malignant lesions [24]. The ADC values depend on many factors such as tissue microstructure, necrosis, presence of macromolecules and also perfusion phenomenon. When compared to benign lesions, abnormal blood perfusion is more prevalent in malignant lesions and ADC values will be affected by both blood perfusion and extracellular space. Unlike some other studies [17], in the current study, we chose three different b factors all over 300 s/mm^2^ since ADC value would better reflect the true water diffusion in the tissue with larger b factors. However, there was no significant difference in ADC value between benign and malignant lesions when the b factors were 500 s/mm^2^ and 800 s/mm^2^. Delormere [25] demonstrated that malignant lesions usually do not have a complete basal membrane of blood vessel which enhances the molecular exchange in the capillary bed. ADC values could be influenced by both blood perfusion and extracellular space. In thyroid malignancy, increased blood perfusion increases the apparent speed of the diffusing water protons while narrow extracellular space will restrict its movement. Although at higher b-values the sensitivity to perfusion is reduced, our results show no difference in ADC values between benign and malignant groups with b-values of 500 s/mm^2^ or 800 s/mm^2^. The ADC values obtained using the lower b-value of 300 s/mm^2^ is most likely affected by both perfusion and diffusion effects. It is possible that the discriminatory effect of this lower 300 s/mm^2^ reflect a combination of altered vascularity and changes in cellular composition that characterizes malignancy.

Fine-needle aspiration biopsy (FNAB) cytology can give 4 different results: benign, malignant, suspicious and non-diagnostic. No definite diagnosis is possible for the two categories of suspicious and non-diagnostic. Although FNAB is commonly used to screen for malignancy, a unique approach in this study is that we used histological results from thyroidectomy as the standard reference for distinguishing benign and malignant lesions. Based on final histological results, we were able to obtain a definite diagnosis of each lesion. ROC curves and cut off point for b-values are more accurate in this approach.

There are still some limitations in this study. First, the relatively small number (33.3%) of the malignant nodules somehow limits the statistical power. This study needs to be expanded further with a larger number of patients. B-values should be extended to lower values and higher ranges to verify that 300 s/mm^2^ is an optimal value. Second, thyroid nodules less than 10 mm were not included in this study. Improvement in the software of diffusion-weighted MR imaging will help in the detection of smaller lesions in future studies. Third, we have not studied the correlation of ADC values with subtypes of thyroid lesions such as cysts from hemorrhagic nodules because of the small number which would reduce its statistical power.

## Conclusion

In conclusion, diffusion-weighted MR imaging is a relatively new and non-invasive approach to assess thyroid lesions. ADC values seem to be able to differentiate benign from malignant thyroid disease. Further studies should be performed to expand the utility of DWI in thyroid lesions.

## Abbreviations

DWI: Diffusion-weighted imaging; ADC: Apparent diffusion coefficient; T1WI: T1 weighted imaging; T2WI: T2 weighted imaging; FNAB: Fine needle aspiration biopsy; ICC: Intraclass correlation coefficient; ROC: Receiver operating characteristic.

## Competing interests

The authors declare that they have no competing interests.

## Authors’ contributions

YW collect data from patients and drafted the manuscript. XY participated in the sequence alignment. WS participated in the sequence alignment. YD did postprocessing and measurement on images. YY did postprocessing and measurement on images. XT conceived of the study, and participated in its design and coordination. CT participated in the design of the study and performed the statistical analysis. All authors read and approved the final manuscript.

## Pre-publication history

The pre-publication history for this paper can be accessed here:

http://www.biomedcentral.com/1471-2342/13/23/prepub
